# Association between perfluoroalkyl acids and liver function: Data on sex differences in adolescents

**DOI:** 10.1016/j.dib.2019.104618

**Published:** 2019-10-05

**Authors:** Roberta Attanasio

**Affiliations:** Georgia State University, USA

**Keywords:** Adolescents, Liver enzymes, NHANES, Perfluoroalkyl acids, Sex differences

## Abstract

The data herein presented show multivariate linear regressions performed to examine the association between individual serum perfluoroalkyl acids (PFAAs) [perfluorooctane sulfonic acid (PFOS); linear form of perfluorooctanoic acid (PFOA); perfluorohexane sulfonic acid (PFHxS); perfluorononanoic acid (PFNA)], and biomarkers of liver function (Sex Differences in the association between perfluoroalkyl compounds and liver function in US adolescents: analyses of NHANES 2013–2016). Data relate to male and female adolescents (ages 12–19 years) who participated to the 2013–2016 National Health and Nutrition Examination Survey. The outcome of interest was represented by changes in biomarkers of liver function, including alanine aminotransferase (ALT), aspartate aminotransferase (AST), and gamma-glutamyl transferase (GGT). Serum measurement values of ALT, AST and GGT were natural log-transformed. Data represent multivariate regression analyses with the single PFAA as β coefficients (and 95%CI), adjusted by age, race/ethnicity, body weight, education, income-to*-*poverty ratio, and exposure to smoking. Single PFAAs were used as continuous natural log-transformed predicted variables in males and females. Analyses were performed also with individuals PFAAs categorized via sex-specific weighted quartile, with cutoffs based on the weighted distribution of the single PFAA in the study population. Because the dependent variables (ALT, AST and GGT) were log-transformed, data were re-transformed by exponentiation of the β coefficients, and presented as percent differences estimated by comparing each of the upper three quartiles to the lowest quartile using the formula 100*(e^β^ −1). Together, these data can serve as a basis to analyze associations between liver function and PFAA exposure taking into account sex differences in adolescent populations.

**Table:** Specifications Table

**Subject**	Environmental Science
**Specific subject area**	Environmental health
**Type of data**	Tables
**How data were acquired**	Downloaded from US National Center for Health Statistics and analyzed using SAS-Callable SUDAAN 10 statistical software
**Data format**	Raw, and analyzed.
**Parameters for data collection**	Restricted to children who participated in the National Health and Nutrition Examination Survey (NHANES) with available environmental chemical biomonitoring and liver enzyme data.
**Description of data collection**	Data collection on sociodemographic characteristics, chemical exposure and serum biomarkers of liver function.
**Data source location**	NHANES—a program of studies designed to assess the health and nutritional status of adults and children in the United States of America conducted by the National Center for Health Statistics (NCHS).
**Data accessibility**	Data are available from the US National Center for Health Statistics. https://wwwn.cdc.gov/nchs/nhanes/ContinuousNhanes/Default.aspx?BeginYear=2011 https://wwwn.cdc.gov/nchs/nhanes/ContinuousNhanes/Default.aspx?BeginYear=2013
**Related research article**	Attanasio R. Sex Differences in the association between perfluoroalkyl acids and liver function in US adolescents: analyses of NHANES 2013–2016. Environ Pollut 2019 Nov; 254(Pt B):113061 https://doi.org/10.1016/j.envpol.2019.113061.

**Table:** 

**Value of the Data** • Data point out currently understudied epidemiological sex differences in human adolescent populations as it relates to liver effects associated with exposure to perfluoroalkyl acids. • By highlighting differences between male and female adolescents in markers of liver function, data can be useful to researchers focusing on the study of pediatric liver disease. • Data provide a basis for further epidemiological analysis of sex differences in different human populations exposed to perfluoroalkyl acids.

## 1 Data

Data on the sex difference association between exposure to perfluoroalkyl acids (PFAAs) and biomarkers of liver function in adolescent (12–19 years old) participants of the National Health and Nutrition Examination Survey (NHANES) 2013–2016 were analyzed [1]. Biomarkers of liver functions as dependent variables included alanine aminotransferase (ALT), aspartate aminotransferase (AST), and gamma glutamyltransferase (GGT). Data for the following PFAAs were analyzed as independent variable: linear form of perfluorooctanoic acid (PFOA); perfluorononanoic acid (PFNA); perfluorohexane sulfonic acid (PFHxS); and, perfluorooctane sulfonic acid (PFOS). Multivariate linear regressions were performed to analyze the data.

Table 1 shows multivariate linear regression adjusted β coefficient (95% CI) by unit of natural log-transformed PFAAs in adolescent participants (ages 12–19 years) in NHANES 2013–2016.

**Table 1:** Multivariate Linear Regression Adjusted^a^ β Coefficient (95% CI) by unit of natural log-transformed perfluoroalkyl acids in adolescent participants (ages 12–19 years) in NHANES 2013–2016.

	Alanine Aminotransferase (ln)		Aspartate Aminotransferase (ln)		Gamma Glutamyltransferase (ln)	
	Male	Female	Male	Female	Male	Female
Perfluorooctanoic acid	−0.07 (−0.13, −0.01)	0.09 (0.02, 0.17)	−0.06 (−0.12, −0.00)	0.06 (−0.00, 0.13)	−0.05 (−0.14, 0.03)	0.10 (0.01, 0.18)
Perfluorononanoic acid	−0.07 (−0.13, −0.02)	0.10 (0.04, 0.17)	−0.07 (−0.10, −0.03)	0.06 (−0.01, 0.13)	−0.06 (−0.13, 0.01)	0.01 (−0.04, 0.06)
Perfluorohexane sulfonic acid	0.01 (−0.03, 0.05)	0.02 (−0.03, 0.07)	−0.01 (−0.05, 0.03)	0.03 (−0.02, 0.07)	0.01 (−0.05, 0.07)	0.02 (−0.03, 0.07)
Perfluorooctane sulfonic acid	0.01 (−0.09, 0.10)	0.09 (−0.01, 0.18)	−0.02 (−0.11, 0.06)	0.07 (0.00, 0.13)	0.05 (−0.04, 0.14)	0.07 (−0.004, 0.14)

a Adjusted by age, race/ethnicity, body weight status (normal/underweight; overweight; obese), education, income-to-poverty ratio, and exposure to smoking.

Table 2 shows the percent differences (95% CI) in serum ALT and serum AST by PFAA levels in adolescent participants (ages 12–19 years) in NHANES 2013–2016.

**Table 2:** Percent Differences (95% CI) in serum Alanine Aminotransferase (ALT) and serum Aspartate Aminotransferase (AST) by perfluoroalkyl acid levels in adolescent participants (ages 12–19 years) in NHANES 2013–2016.

	Alanine Aminotransferase^a^		Aspartate Aminotransferase^a^	
	Males	Females	Males	Females
Perfluorooctanoic acid				
Quartile 1	Referent	Referent	Referent	Referent
Quartile 2	2.02 (−14.79, 20.92)	9.42 (1.01, 19.72)	−1.00 (−13.93, 12.75)	4.08 (−1.98, 11.63)
Quartile 3	−1.00 (−12.19, 10.52)	17.35 (5.13, 32.31)	−0.40 (−7.69, 8.33)	10.52 (1.01, 20.92)
Quartile 4	−10.42 (−18.94,-1.00)	18.53 (5.13, 32.31)	−4.88 (−13.93, 4.08)	11.63 (1.01, 22.14)
Perfluorononanoic acid				
Quartile 1	Referent	Referent	Referent	Referent
Quartile 2	−12.19 (−24.42, 3.05)	4.08 (−6.76, 15.03)	−8.61 (−16.47, −0.40)	1.01 (−7.69, 10.52)
Quartile 3	−13.06 (−25.92, 3.05)	8.33 (−2.96, 19.72)	−2.96 (−11.31, 6.18)	8.33 (−1.00, 18.53)
Quartile 4	−13.93 (−25.17, −0.49)	17.35 (6.18, 31.00)	−11.31 (−18.13, 4.08)	10.52 (0.40, 22.14)
Perfluorohexane sulfonic acid				
Quartile 1	Referent	Referent	Referent	Referent
Quartile 2	−6.76 (−13.93, 1.01)	−1.00 (−13.06, 12.75)	−3.92 (−9.52, 10.52)	−0.40 (−9.52, 10.52)
Quartile 3	−8.61 (−17.30, 2.02)	5.13 (−4.88, 17.35)	−2.96 (−8.61, 4.08)	7.25 (−1.00, 16.18)
Quartile 4	−1.98 (−11.31, 8.33)	3.05 (−9.52, 17.35)	0.40 (−8.61, 9.42)	3.05 (−7.69, 15.03)
Perfluorooctane sulfonic acid				
Quartile 1	Referent	Referent	Referent	Referent
Quartile 2	−4.88 (−18.94, 11.63)	−1.98 (−15.63, 15.03)	−1.98 (−10.42, 8.33)	3.05 (−7.69, 15.03)
Quartile 3	7.25 (−4.88, 20.92)	1.01 (−10.42, 13.88)	1.01 (−6.76, 10.52)	5.13 (−3.92, 13.88)
Quartile 4	−1.00 (−13.06, 13.88)	11.63 (−1.98, 27.12)	−1.00 (−11.31, 10.52)	12.75 (3.05, 23.37)

a Adjusted by age, race/ethnicity, body weight status (normal/underweight; overweight; obese), education, income-to-poverty ratio, and exposure to smoking.

Table 3 shows the percent differences (95% CI) in serum GGT by PFAA levels in adolescent participants (ages 12–19 years) in NHANES 2013–2016.

**Table 3:** Percent Differences (95% CI) in serum Gamma Glutamyltransferase (GGT) by perfluoroalkyl acid levels in adolescent participants (ages 12–19 years) in NHANES 2013–2016.

	Gamma Glutamyltransferase^a^	
	Males	Females
Perfluorooctanoic acid		
Quartile 1	Referent	Referent
Quartile 2	1.01 (−6.76, 8.33)	10.52 (3.05, 18.53)
Quartile 3	−2.96 (−12.19, 7.25)	19.72 (5.13, 34.99)
Quartile 4	−12.19 (−22.12, 0.40)	18.53 (3.05, 34.99)
Perfluorononanoic acid		
Quartile 1	Referent	Referent
Quartile 2	−1.98 (−14.79, 11.63)	4.08 (−6.76, 16.18)
Quartile 3	−6.76 (−16.47, 4.08)	8.33 (−6.76, 25.86)
Quartile 4	−8.61 (−21.34, 6.18)	2.02 (−8.61, 12.75)
Perfluorohexane sulfonic acid		
Quartile 1	Referent	Referent
Quartile 2	−8.61 (−18.94, 3.05)	10.52 (1.01, 22.14)
Quartile 3	−2.96 (−13.93, 9.42)	10.52 (−1.00, 22.14)
Quartile 4	2.02 (−11.31, 16.18)	8.33 (−1.98, 19.72)
Perfluorooctane sulfonic acid		
Quartile 1	Referent	Referent
Quartile 2	−1.98 (−11.31, 9.42)	7.25 (−5.82, 22.14)
Quartile 3	−1.00 (−10.42, 9.42)	7.25 (−1.98, 17.35)
Quartile 4	4.08 (−9.52, 19.72)	12.75 (1.01, 24.61)

a Adjusted by age, race/ethnicity, body weight status (normal/underweight; overweight; obese), education, income-to-poverty ratio, and exposure to smoking.

## 2 Experimental design, materials, and methods

NHANES (https://www.cdc.gov/nchs/nhanes/index.htm) is a cross-sectional, nationally representative survey of the non-institutionalized civilian population of the United States conducted annually by CDC's National Center for Health Statistics (CDC/NCHS) [2]. The survey employs a multistage stratified probability sample based on selected counties, blocks, households, and persons within households. The data from publicly available files for NHANES cycles 2013–2014 and 2015–2016 were merged using NCHS recommendations [2]. Data on the sex difference association between exposure to perfluoroalkyl acids (PFAAs) and biomarkers of liver function in adolescent (12–19 years old) participants of the National Health and Nutrition Examination Survey (NHANES) 2013–2016 were analyzed. Biomarkers of liver functions as dependent variables included alanine aminotransferase (ALT), aspartate aminotransferase (AST), and gamma glutamyltransferase (GGT). Data for the following PFAAs were analyzed as independent variable: linear form of perfluorooctanoic acid (PFOA); perfluorononanoic acid (PFNA); perfluorohexane sulfonic acid (PFHxS); and, perfluorooctane sulfonic acid (PFOS). Multivariate linear regressions were performed to analyze the data.

Serum PFAAs were measured using automated solid-phase extraction along with reverse-phase high-performance liquid chromatography/tandem mass spectrometry by CDC's National Center for Environmental Health (NCEH), Division of Laboratory Sciences (DLS). Detailed methodology and QA/QC instructions are discussed in the NHANES Laboratory Procedures Manual (https://wwwn.cdc.gov/nchs/data/nhanes/2013-2014/labmethods/PFAS_H_MET.pdf). NCEH/DLS analyzed the serum levels of fourteen different PFASs.

The present analyses are based on the exposure data of four PFAAs: PFOA (linear isomers), PFNA, PFOS (both linear and branched isomers) and PFHxS, which were detected in 95% of the samples. To obtain total PFOS, concentrations of the branched and linear isomers were summed. The limit of detection (LOD) was 0.1 ng/mL for all four PFAAs included in the analysis. For concentrations less than the LOD, a value equal to the limit of detection divided by the square root of two was used.

After collection by NCHS–trained professionals, the serum specimens were refrigerated and shipped to a central laboratory for analysis. The Collaborative Laboratory Services used a Beckman Synchron LX20 analyzer to measure the biochemistry profile, including levels alanine aminotransferase (ALT), aspartate aminotransferase (AST), and gamma glutamyltransferase (GGT). ALT, AST and GGT were not normally distributed, thus they were natural log-transformed in the analyses. Participants were excluded if: 1) they had been told by a doctor or other health professional that they had hepatitis B or hepatitis C; 2) were positive for hepatitis B core antibody, and 3) were positive for hepatitis E IgG or IgM antibodies.

The following covariates were considered in analyses: sex, age, race/ethnicity, obesity, income*-*to-poverty ratio (PIR), and exposure to tobacco. Race/ethnicity was categorized as “non-Hispanic white,” “non-Hispanic black,” “Hispanic,” “non-Hispanic Asian,” and “other race and multiracial.” Children and adolescents were classified as underweight, normal weight, overweight, or obese according to age and sex, based on the Centers for Disease Control and Prevention's sex-specific 2000 BMI-for-age growth charts for the United States. The BMI categories were provided in the NHANES public examination files (http://wwwn.cdc.gov/Nchs/Nhanes/2013-2014/BMX_H.htm, and https://wwwn.cdc.gov/Nchs/Nhanes/2015-2016/BMX_I.htm); participants in underweight and normal weight categories were combined in analyses. Income*-to-*poverty ratio (PIR) is a measure of socioeconomic status and represents the calculated ratio of household income to the poverty threshold after accounting for inflation and family size, with income values < 1 representing those below the poverty line. Smoking exposure was defined based upon information collected in the NHANES questionnaire on household smoking and information about self-reported use of tobacco products during the previous 5 days. Therefore, exposure to smoking was categorized in: 1) no exposure to smoking, consisting of participants who did not report use of tobacco products in the previous five days and lived in a household where no one was a smoker; 2) exposure to smoking, consisting of participants who reported use of tobacco products in the previous 5 days, or lived in a household where someone was a smoker. For adolescents (12–19 years of age), the questions on tobacco use were self-administered using the Audio Computer-Assisted Self-Interview system.

All analyses were performed using PFAA-specific subsample weight as recommended by NCHS, to account for the complex sampling design and non-response of NHANES. The statistical software SAS 9.4 (SAS Institute, Cary, NC) and SAS-Callable SUDAAN 10 (Research Triangle Institute, Research Triangle Park, NC) were used to account for the NHANES complex sample design. Multivariate linear regression was used to calculate adjusted β-coefficients and 95% confidence intervals (CIs) for the associations between liver biomarkers with serum PFAA levels. Each individual PFAA compound was employed in the models as a continuous natural log-transformed (ln) variable. Analyses of the individual compounds — PFOA (linear isomer), PFOS, PFNA, and PFHxS — were also done as categorized via sex-specific weighted quartiles, with cutoffs based on the weighted distribution of the single PFAA in the study population. The range value of weighted distributed quartiles were the following for males: 1) Linear-PFOA: Q1≤1.10 ng/mL; Q2 = 1.11–1.48 ng/mL; Q3 = 1.49–1.90 ng/mL; Q4>1.90 ng/mL; 2) PFNA: Q1≤0.36 ng/mL; Q2 = 0.37–0.50 ng/mL; Q3 = 0.51–0.74 ng/mL; Q4>0.74 ng/mL; 3) PFHxS: Q1≤0.72 ng/mL; Q2 = 0.73–1.09 ng/mL; Q3 = 1.10–2.05 ng/mL; Q4>2.05 ng/mL. 4) PFOS: Q1≤2.60 ng/mL; Q2 = 2.61–3.70 ng/mL; Q3 = 3.71–5.41 ng/mL; Q4> 5.41 ng/mL. The range value of weighted distributed quartiles for females were the following: 1) Linear-PFOA: Q1≤0.79 ng/mL; Q2 = 0.80–1.13 ng/mL; Q3 = 1.14–1.67 ng/mL; Q4>1.67 ng/mL; 2) PFNA: Q1≤0.27 ng/mL; Q2 = 0.28–0.43 ng/mL; Q3 = 0.44–0.67 ng/mL; Q4>0.67 ng/mL; 3) PFHxS: Q1≤0.47 ng/mL; Q2 = 0.48–0.75 ng/mL; Q3 = 0.76–1.40 ng/mL; Q4>1.40 ng/mL; 4) PFOS: Q1≤1.80 ng/mL; Q2 = 1.81–2.65 ng/mL; Q3 = 2.66–4.14 ng/mL; Q4> 4.14 ng/mL.

Because the dependent variables (ALT, AST and GGT) were log-transformed, results were re-transformed by exponentiation of the β coefficients, and also presented as percent differences estimated by comparing each of the upper three quartiles to the lowest quartile using the formula 100*(e^β^ −1).
